# The Effects of Breast Cancer Surgery on Thoracic Kyphosis and the Correlation Between Supine CT and Standing X-Rays: A Single-Center Retrospective Study

**DOI:** 10.3390/jcm15155951

**Published:** 2026-07-30

**Authors:** Sejoon Kim, Ho-Geon Namgung, Ah Yeon Lee, Kyung Hyun Park, Jong In Lee

**Affiliations:** Department of Rehabilitation Medicine, Seoul St. Mary’s Hospital, College of Medicine, The Catholic University of Korea, Seoul 06591, Republic of Korea

**Keywords:** breast neoplasms, mastectomy, breast-conserving surgery, spinal curvatures, kyphosis

## Abstract

**Background/Objectives:** Breast cancer surgery can induce postural adaptations that influence spinal alignment. Although previous studies have predominantly addressed coronal deformities such as scoliosis, changes in sagittal alignment, particularly thoracic kyphosis, remain poorly characterized. An increased kyphotic angle has been associated with impaired physical function, emphasizing the importance of assessing sagittal spinal changes after surgery. Although thoracic kyphosis is traditionally measured on standing radiographs, it can also be evaluated using supine chest computed tomography (CT), routinely obtained during postoperative follow-up. This study investigated the impact of breast cancer surgery on thoracic kyphotic angle and assessed the correlation between kyphotic angles measured on supine CT and standing X-rays. **Methods:** This retrospective study included 185 breast cancer patients who underwent multiple chest CT and whole-spine X-rays. The thoracic kyphotic angle was defined as the Cobb angle from T4 to T12 on sagittal images. Changes in kyphotic angle among the three surgical groups were analyzed using linear mixed-effects models adjusted for age, chemotherapy, radiotherapy, and endocrine treatment. The correlation between supine CT and standing X-ray was analyzed using Pearson’s coefficient. **Results:** The mean follow-up interval was 42.6 ± 6.3 months. The mean angular change was minimal (0.6 ± 2.8°), and no significant effects of time, surgical group, or their interaction were observed. Kyphotic angles measured on standing X-rays strongly correlated with those obtained from supine CT (r = 0.799, *p* < 0.001), with standing values approximately 7.4° higher. **Conclusions:** Breast cancer surgery did not significantly alter the thoracic kyphotic angle. Supine CT provides a reliable and practical alternative for the assessment of postoperative kyphosis during follow-up.

## 1. Introduction

Breast cancer is among the most common cancers in women. With the availability of effective treatment strategies, the 5-year survival rate for breast cancer patients in the United States is approximately 89%, and the 10-year survival rate is 83% [1].

Breast cancer surgery has evolved substantially in recent years, with a marked shift toward de-escalation strategies and a broadening range of surgical approaches, from breast-conserving surgery to mastectomy with or without immediate reconstruction [2]. Various physiological changes following surgical management, such as breast-conserving surgery (BCS) or mastectomy-related breast asymmetry, pain, or limited upper limb mobility, can lead to adaptive postural changes [3,4,5]. Some studies have reported that unilateral mastectomy may induce or exacerbate scoliosis [6,7,8]. Conversely, other studies have shown that breast cancer surgery does not adversely affect coronal spinal alignment [9].

In healthy adults, the thoracic kyphotic angle increases with age: the Cobb angle averages approximately 20–29° in younger adults and rises to a mean of 35–38° in those aged 65 years and older [10]. Although a universally accepted threshold distinguishing normal kyphosis from hyperkyphosis is lacking, a Cobb angle of 50° in the standing position has been used to define hyperkyphosis in several studies [10]. A recent study demonstrated that a larger kyphotic angle was independently associated with poorer physical performance both at baseline and over time. These findings underscore the importance of early detection and management of hyperkyphosis to prevent further progression and potentially preserve physical function [11]. However, research on spinal alignment in breast cancer patients has largely focused on coronal deformities, such as scoliosis, with few studies addressing sagittal alignment, including thoracic kyphosis [12]. Consequently, it is important to evaluate thoracic kyphotic angles and to identify factors that contribute to kyphotic changes in this patient population.

Scoliosis is typically assessed in the standing position; however, in some cases, the Cobb angle can also be measured in the supine position. Previous studies have demonstrated a strong correlation between Cobb angles obtained from supine and standing radiographs [13], with standing measurements generally 5–10° higher than those obtained in the supine position [13,14,15]. Nevertheless, few studies have investigated the correlation between kyphotic angle measurements in the supine and standing positions [16].

In breast cancer patients, chest computed tomography (CT) is routinely performed in the supine position to monitor for cancer recurrence. If kyphotic angles measured on supine CT show a strong correlation with those obtained in the standing position, it would be feasible to assess changes in thoracic kyphotic angle using routinely acquired CT scans.

Therefore, this study evaluated the impact of breast cancer surgery on the thoracic kyphotic angle and examined the correlation of kyphotic measurements between the supine and standing positions.

## 2. Materials and Methods

### 2.1. Patient Population

Patients were identified from the clinical data warehouse (CDW) at Seoul St. Mary’s Hospital between 1 September 2017 and 31 August 2022, with all clinical information obtained from electronic medical records.

This retrospective study included patients diagnosed with breast cancer between 1 September 2017 and 31 August 2019 who had an initial preoperative chest CT and a postoperative whole-spine lateral standing X-ray during the same period and had undergone postoperative chest CTs between 1 September 2019 and 31 August 2022. Standing whole-spine radiographs were not routinely obtained in all postoperative breast cancer patients. They were performed at a single, early postoperative point in patients who raised concerns about postural changes or spinal alignment after surgery.

Using the CDW, we initially identified 250 patients who met the inclusion criteria. Patients were then excluded if they met any of the following exclusion criteria: absence of a preoperative chest CT performed at our hospital; a follow-up period of less than 2 years between the initial and last CT; additional surgical procedures for recurrence; compression fractures at T4–T12; inability to measure the Cobb angle due to missing sagittal views or inadequate visualization of T4–T12; prior breast cancer surgery; spinal bone metastasis confirmed by bone scan; no breast cancer surgery performed; or bilateral breast cancer surgery. Based on these criteria, 65 patients were excluded, resulting in a total of 185 patients being included in the analysis.

**Figure 1:**
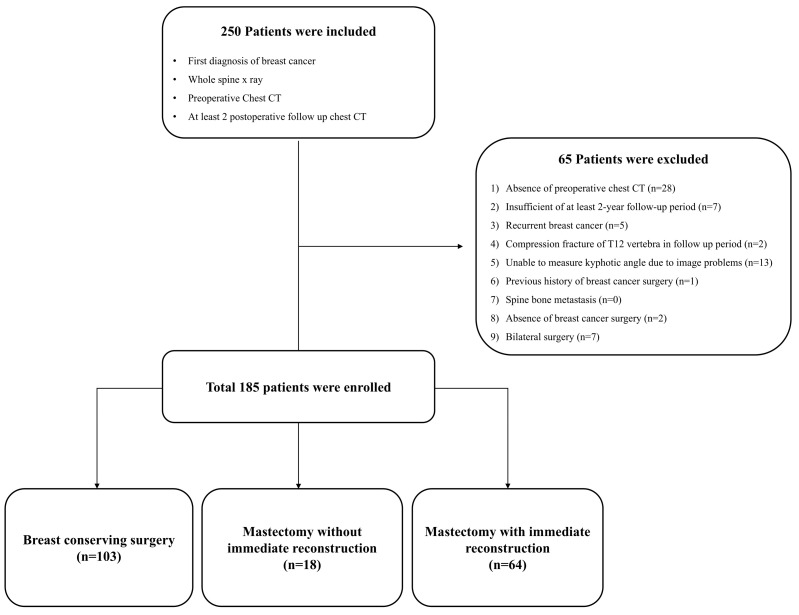
Flow diagram depicting the study’s patient selection process.

Patients were further categorized into three groups according to the type of breast cancer surgery: BCS, mastectomy with immediate reconstruction (IRB), and mastectomy without reconstruction (MA). The process of patient selection is summarized in the flowchart presented in Figure 1.

### 2.2. Data Collection and Definition

We collected demographic and clinical data that could potentially influence spinal alignment. The variables included age, body mass index (BMI), presence of osteoporosis, and details of breast cancer treatment, including surgery type, chemotherapy, radiation therapy, and hormone therapy. BMI was calculated as weight in kilograms divided by height in meters squared (kg/m^2^). Osteoporosis was confirmed using bone mineral density measurements obtained by dual-energy X-ray absorptiometry (Horizon W densitometer; Hologic, Inc., Marlborough, MA, USA). Bone mineral density was assessed as part of the routine baseline oncologic work-up, and osteoporosis status was determined at baseline before longitudinal follow-up.

The thoracic kyphotic angle was measured as the Cobb angle between the upper border of T4 and the lower border of T12 on sagittal CT or X-ray images. Unlike whole-spine X-rays, CT provides multiple sagittal views; the view closest to the midline of the body and clearly delineating T4 and T12 was selected for measurement. The Cobb angle was determined by drawing a line along the upper border of the upper vertebra and another along the lower border of the lower vertebra, then measuring the angle formed at the intersection of the perpendiculars to these lines. Angle calculations were performed automatically using a tool integrated within the picture archiving and communication system. Kyphotic angles were measured four times: on preoperative chest CT, on the earliest postoperative whole-spine X-ray and chest CT, and on the most recent chest CT within the follow-up period, with a minimum interval of 2 years from the initial measurement. Angular change was defined as the difference between the last and initial CT measurements. Based on previous studies of spinal kyphosis [15,16], a kyphotic angular change greater than 5° was predefined as clinically significant.

To evaluate measurement reliability, both intraobserver and interobserver variabilities in the thoracic kyphotic angle were analyzed. Intraobserver variability was assessed by having clinician 1 (H.G.NG) measure the Cobb angle on 50 randomly selected radiographs, with a second measurement performed 2 weeks later. Interobserver variability was assessed by clinician 2 (S.J.K), who was blinded to clinician 1’s measurements, measuring the thoracic kyphotic angle on the same initial and last follow-up chest CT images. Measurements from clinician 2 were then compared with the first set obtained by clinician 1. Both intra- and interobserver reliabilities were quantified using intraclass correlation coefficients (ICCs) based on a two-way mixed-effects, random-effects model with absolute agreement.

### 2.3. Statistical Analyses

Baseline demographic and clinical characteristics are summarized as mean ± standard deviation for continuous variables and as counts and percentages for categorical variables. The Shapiro–Wilk test was used to assess the normality of baseline characteristics and kyphotic angle values. Group differences were evaluated using one-way analysis of variance with Tukey’s honestly significant difference post hoc test, the Kruskal–Wallis test, chi-square test, or Fisher’s exact test, as appropriate. Changes in thoracic kyphotic angle over time and the interaction between time and surgical group were analyzed using linear mixed-effects models (LMMs), adjusting for age, chemotherapy, radiotherapy, and endocrine treatment. A *p*-value < 0.05 was considered statistically significant.

Pearson’s correlation coefficient was used to assess the relationship between kyphotic angles measured on whole-spine lateral X-rays and chest CT obtained at the earliest postoperative time point. Linear regression analysis was further performed to derive the equation describing the relationship between these two measurements.

All statistical analyses were conducted using RStudio for Windows (version 2025.09.1; Posit Software, PBC, Boston, MA, USA) and SPSS Statistics for Windows (version 24.0; IBM Corp., Armonk, NY, USA), with statistical significance defined as *p* < 0.05.

## 3. Results

### 3.1. Demographic and Clinical Characteristics

In total, 185 patients were included in the analysis: 103 in the BCS group, 18 in the MA group, and 64 in the IRB group. The IRB group consisted of patients who underwent implant-based reconstruction (*n* = 26) or autologous flap reconstruction (*n* = 38), the latter including 37 deep inferior epigastric perforator flaps and one latissimus dorsi flap. Baseline characteristics are summarized in Table 1. The mean age was 54.8 ± 10.2 years, and the mean BMI was 23.4 ± 3.6 kg/m^2^. Patients in the MA group were significantly older than those in the BCS and IRB groups (*p* < 0.001), whereas baseline BMI did not differ significantly among groups (*p* = 0.694). The distribution of lymph node surgery types varied significantly across groups, with sentinel lymph node biopsy being most common in the BCS group, whereas axillary dissection was more frequent in the MA and IRB groups (*p* < 0.001). The proportions of patients receiving chemotherapy, radiotherapy, and endocrine therapy also differed significantly among surgical groups (all *p* < 0.05). The prevalence of osteoporosis and bone metastasis did not differ significantly between groups.

### 3.2. The Thoracic Kyphotic Angle Measurements and Longitudinal Change

The thoracic kyphotic angle measurements demonstrated excellent reliability between the two clinicians. Intraobserver ICCs were 0.968 (95% confidence interval [CI], 0.94–0.98) for the initial CT scan and 0.947 (95% CI, 0.91–0.97) for the last follow-up CT scan. Interobserver ICCs were 0.957 (95% CI, 0.943–0.968) for the initial CT scan and 0.950 (95% CI, 0.934–0.963) for the last follow-up CT scan.

At baseline chest CT, the mean thoracic kyphotic angle across all patients was 25.9 ± 6.6°, with no significant differences among surgical groups (*p* = 0.748). At the first follow-up CT, the mean angle was 25.4 ± 6.6°, and at the last follow-up CT, it was 26.5 ± 6.3°, with no significant differences between groups at either time point (*p* = 0.300 and *p* = 0.714, respectively). The mean angular change over the follow-up period was minimal (0.6 ± 2.8°), and only 10 patients (5.2%) exhibited a change exceeding the predefined threshold for significance. The mean interval between the initial and last follow-up CT was 42.6 ± 6.3 months, which did not differ across surgical groups (Table 2).

**Figure 2:**
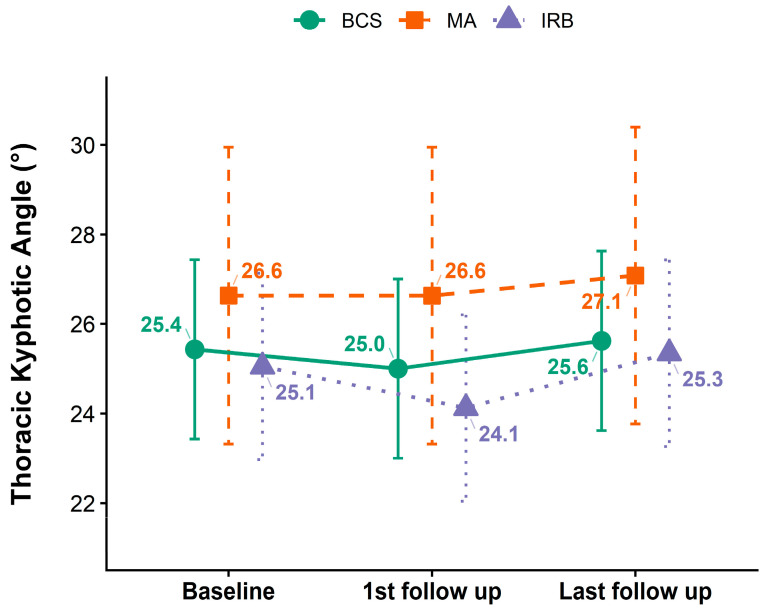
Estimated marginal means of thoracic kyphotic angles across time points.

LMMs further supported these findings. After adjusting for age, chemotherapy, radiotherapy, and endocrine treatment, LMM analysis revealed no significant main effects of time, surgical group, or time × group interaction on thoracic kyphotic angle. In the adjusted LMM, the estimated baseline kyphotic angle was 23.8°, with no significant longitudinal changes observed during follow-up (first follow-up vs. baseline: −0.43°, *p* = 0.12; last follow-up vs. baseline: +0.19°, *p* = 0.50). Surgical type was not associated with baseline kyphotic angle (IRB: −0.38°, *p* = 0.77; MA: +1.20°, *p* = 0.52), and no significant interaction between time and surgical type was detected (Type III Wald χ^2^ = 2.88, df = 4, *p* = 0.578; all individual interaction terms *p* ≥ 0.27) (Figure 2).

Linear mixed-effects model estimates (mean ± 95% confidence intervals) of thoracic kyphotic angles at baseline, first follow-up, and last follow-up, according to surgical groups. Lines connect the model-predicted means, and error bars represent 95% confidence intervals.

### 3.3. The Thoracic Kyphotic Angles in the Supine and Standing Positions

**Figure 3:**
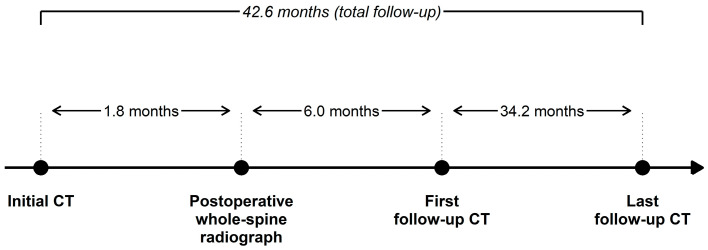
Timeline of follow-up imaging. Schematic illustration of the imaging schedule, showing intervals between the initial CT and whole-spine radiograph, between the radiograph and the first follow-up CT, and between the first and last follow-up CT.

**Figure 4:**
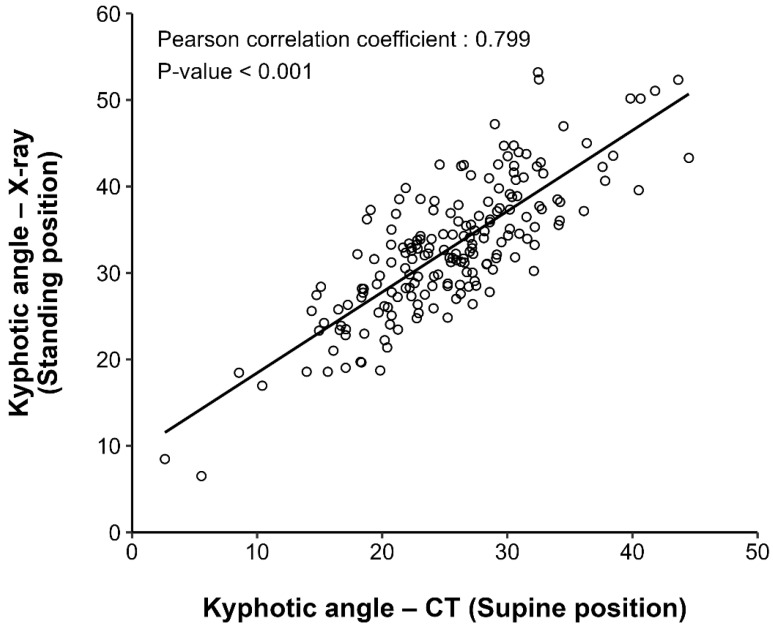
Linear regression model predicting standing kyphotic angle. Regression line demonstrating the relationship between standing and supine thoracic kyphotic angles, with the regression equation and coefficient of determination (R^2^) shown.

The mean thoracic kyphotic angle measured on whole-spine standing radiographs was 32.9 ± 7.7°, compared with 25.9 ± 6.6° measured on supine CT at the corresponding time point (Table 2). The mean interval between the two imaging studies was 6.0 ± 5.6 months (Figure 3). A strong correlation was observed between kyphotic angles measured on CT (supine) and X-ray (standing) (Pearson’s r = 0.799, *p* < 0.001), with standing measurements approximately 7.4° higher (Figure 4). Linear regression analysis produced the following predictive equation:

Standing kyphotic angle = 0.935 × supine kyphotic angle + 9.09 (R^2^ = 0.637) (1)

## 4. Discussion

This study examined the long-term impact of breast cancer surgery on the thoracic kyphotic angle in patients with breast cancer. Over a mean follow-up period of 42.6 months, the mean kyphotic angular change was minimal (0.6 ± 2.8°) and within the known measurement variability for spinal sagittal parameters. Importantly, this magnitude of change is below the threshold generally considered clinically meaningful, indicating that postoperative alterations in thoracic kyphosis were negligible from a clinical perspective. Additionally, surgical type was not significantly associated with changes in kyphotic angle.

Previous studies have primarily focused on changes in spinal alignment, particularly scoliosis, before and after breast cancer surgery. For example, Serel et al. [6] reported that at 1 year after unilateral mastectomy, 38 of 60 patients exhibited an increase in the Cobb angle, with 2 patients demonstrating an increase of 10° or more, resulting in a diagnosis of scoliosis.

Gutkin et al. [7] reported that in breast cancer patients, the Cobb angles measured before mastectomy and at 5 years post-mastectomy differed significantly, with an average increase of 4.7° compared to the initial measurement. Conversely, Nam et al. [9] found that breast cancer surgery did not significantly affect the Cobb angle, with observed differences attributed to measurement variability. Furthermore, surgical type was not associated with changes in the Cobb angle.

Hyperkyphosis has multiple clinical implications. It is associated with reduced physical performance and an increased risk of falls due to a forward shift in the center of gravity, which impairs balance [10,11,17]. In women with an average age of 71, hyperkyphosis has been shown to increase pressure on the anterior vertebral bodies, elevating the risk of vertebral fractures [18]. Additionally, hyperkyphosis has been linked to increased mortality in patients with end-stage renal disease (ESRD) [19]. Collectively, these studies highlight the associations of hyperkyphosis with physical function, fall risk, vertebral fractures, and mortality. Early detection and intervention are therefore clinically important to prevent the progression of kyphotic deformity.

In breast cancer patients, various adjuvant treatments may influence spinal alignment in addition to surgical factors such as mastectomy-related breast asymmetry, pain, or limited upper limb mobility. Postoperative breast asymmetry may alter the anterior weight distribution and shift the center of pressure, potentially leading to compensatory postural adaptations and changes in spinal alignment. Radiotherapy can reduce the flexibility and mobility of the skin and subcutaneous tissues, whereas hormonal therapies may negatively affect bone quality. Chemotherapy can also substantially impact bone health by inducing premature menopause and directly altering bone turnover [20].

Tanrıverdi et al. [12] reported a statistically significant increase in the thoracic kyphotic angle from 33.60 ± 2.97° at baseline to 36.19 ± 4.62° at 2 years after breast cancer surgery. Although statistically significant, this change (approximately 2.6°) was relatively small and may fall within the range of measurement error or the minimal detectable difference for Cobb angle measurements, implying limited clinical relevance. Furthermore, in a subgroup of 12 patients followed for 5 years, no significant change from baseline was observed [12].

In our study, which included 185 patients with a mean follow-up of 42 months, the mean angular change in the thoracic kyphotic angle was only 0.6 ± 2.8°. No statistically significant changes in kyphotic angle were observed over time, and surgical type did not significantly influence the results.

Conversely, Tanrıverdi et al. [12] reported a greater increase in kyphotic angle in a BCS group (*n* = 8) compared with a modified radical mastectomy group (*n* = 49) (4.12° ± [SD] vs. 2.35 ± 2.24°, *p* = 0.048). The absence of significant differences among surgical groups in our study may be explained by the substantially larger sample size in the BCS group (*n* = 103), which provides more robust estimates and reduces the likelihood of sampling bias.

Consistent with our findings, Takeda et al. [21] reported no significant change in the thoracic kyphotic angle between baseline and final follow-up in 53 subjects aged 50 years or older with no history of vertebral fracture. Over a follow-up period exceeding 10 years, thoracic kyphosis measured 27.0 ± 12.8° at baseline and 28.7 ± 16.1° at the final follow-up. It should be noted, however, that their study included healthy individuals without breast cancer, in contrast to our patient population.

We also evaluated the correlation between thoracic kyphotic angle measurements in the supine and standing positions. The two measures were strongly correlated, with standing measurements averaging 7.4° higher than those obtained in the supine position. Using linear regression, we derived an equation to predict the standing kyphotic angle from the supine measurement.

In the evaluation of scoliosis, the Cobb angle is typically measured on radiographs obtained in the standing position. However, in some cases, Cobb angles can also be assessed using imaging studies performed in the supine position, such as CT or MRI. Previous studies have demonstrated a correlation between Cobb angles measured in the supine and standing positions, with standing measurements generally being larger [13,14,15,16].

For example, Vavruch et al. [13] measured Cobb angles in 128 patients with late-onset scoliosis using both supine and standing imaging. The average difference between positions was 11°, with standing measurements averaging 59° and supine measurements 48°, showing a significant correlation. Similarly, Lee et al. [15] evaluated 70 adolescent idiopathic scoliosis patients using both X-ray and MRI and found a significant correlation and an average positional difference of approximately 10°. However, few studies have investigated the correlation of kyphotic angles between supine and standing positions. Benlong et al. [16] measured thoracic kyphosis in 52 adolescent idiopathic scoliosis patients using X-ray and MRI and reported mean kyphotic angles of 16.3 ± 9.1° in the standing position and 11.8 ± 6.1° in the supine position.

We observed a strong correlation between thoracic kyphotic angles measured in the supine and standing positions, with standing measurements averaging approximately 7.4° higher. This finding implies that previously acquired chest CT scans can be used to assess the thoracic kyphotic angle, potentially reducing the need for additional standing X-ray imaging.

This study has several limitations. First, the number of patients in the MA group was relatively small. Second, although the mean follow-up period was 42 months, this duration may be insufficient to detect long-term changes (>5–10 years) in the kyphotic angle, and longer follow-up in a larger cohort is warranted. Third, an important limitation of this study is the absence of data regarding breast size or reconstruction volume. From a biomechanical perspective, the weight and volume of breast tissue can significantly influence the center of gravity and degree of thoracic kyphosis. As this variable was not controlled for in our statistical model, it remains possible that breast size may have confounded the relationship between surgical type and the kyphotic angle. Future studies should aim to incorporate breast volume or mastectomy weight as covariates to isolate more accurately the biomechanical effects of different surgical procedures on spinal alignment. Fourth, CT and standing radiographs were not performed on the same day. However, whole-spine standing radiographs were obtained within 2 months of the initial CT, a timeframe unlikely to reflect meaningful structural or postural change in the thoracic spine. Furthermore, longitudinal CT analysis demonstrated minimal change in the kyphotic angle over time, with nearly identical values at the first follow-up CT (8 months) and a mean change of only 0.6° over a mean follow-up period of 42.6 months, which falls within the known measurement variability for sagittal spinal parameters. This long-term biomechanical stability of the thoracic spine supports the conclusion that the brief timing differences between imaging modalities did not materially influence the study results. Finally, not all imaging studies underwent double assessment. To address this limitation, we evaluated intra- and interobserver variabilities for the first and last CT scans and confirmed excellent reliability. Finally, because standing whole-spine radiographs were not routinely obtained in all postoperative breast cancer patients, the study population represents a selected subgroup who raised concerns about postural changes or spinal alignment after surgery, which may have introduced selection bias and limited the generalizability of our findings. However, longitudinal changes in thoracic kyphosis were assessed using serial chest CT scans obtained during routine oncologic surveillance rather than repeated standing radiographs. As these follow-up CT scans were acquired independently of patient-reported postural concerns, the potential impact of the initial patient selection on the longitudinal assessment is likely to have been minimized.

## 5. Conclusions

Breast cancer surgery, regardless of surgical type, was not associated with clinically significant changes in thoracic kyphotic angle. In addition, thoracic kyphotic angles measured on supine chest CT showed a strong correlation with those measured on standing whole-spine radiographs. These findings suggest that routine chest CT may serve as a practical alternative for longitudinal assessment of thoracic sagittal alignment in breast cancer survivors, potentially reducing the need for additional radiographic examinations.

## Figures and Tables

**Table 1:** Demographic and clinical features of patients.

Characteristics	Total (*n* = 185)	BCS (*n* = 103)	MA (*n* = 18)	IRB (*n* = 64)	*p* Value
Age (years)	54.8 ± 10.2	55.5 ± 9.9	63.6 ± 12.6	51.3 ± 8.1	<0.001 *
BMI (kg/m^2^)	23.4 ± 3.6	23.2 ± 3.7	23.9 ± 3.6	23.5 ± 3.5	0.694
Types of lymph node surgery					<0.001 *
Sentinel lymph node biopsy; *n* (%)	130 (70.3%)	88 (85.4%)	10 (55.6%)	32 (50%)	
Axillary lymph node dissection; *n* (%)	55 (29.7%)	15 (14.6%)	8 (44.4%)	32 (50%)	
Chemotherapy; *n* (%)	129 (69.7%)	63 (61.2%)	14 (77.8%)	52 (81.2%)	<0.001 *
Radiotherapy; *n* (%)	134 (72.4%)	97 (94.2%)	8 (44.4%)	29 (45.3%)	<0.001 *
Endocrine treatment; *n* (%)	155 (83.8%)	90 (87.4%)	12 (66.7%)	53 (82.8%)	<0.001 *
Osteoporosis; *n* (%)	57 (30.8%)	34 (33.0%)	8 (44.4%)	15 (23.4%)	0.179
Bone metastasis; *n* (%)	1 (0.5%)	0 (0.0%)	0 (0.0%)	1 (1.6%)	0.387

Data are expressed as mean ± standard deviation (SD) or number (%). Group comparisons were performed using one-way analysis of variance with Tukey post hoc test, Kruskal–Wallis test, chi-square test, or Fisher exact test, as appropriate. * *p* < 0.05 indicates statistical significance. Abbreviations: ANOVA = analysis of variance; BCS = breast-conserving surgery; BMI = body mass index; IRB = immediate reconstruction after mastectomy; MA = mastectomy alone; SD = standard deviation.

**Table 2:** Thoracic kyphotic angles at each time point and imaging intervals.

	Total (*n* = 185)	BCS (*n* = 103)	MA (*n* = 18)	IRB (*n* = 64)	*p*-Value
Kyphotic angle by CT (supine), °					
Initial CT	25.9 ± 6.6	25.9 ± 6.6	26.9 ± 7.0	25.6 ± 6.6	0.748
1st follow-up CT	25.4 ± 6.6	25.6 ± 6.5	27.1 ± 7.3	24.7 ± 6.6	0.300
Last follow-up CT	26.5 ± 6.3	26.4 ± 6.4	27.7 ± 6.6	26.3 ± 6.2	0.714
Angular change (Δ, °)	0.6 ± 2.8	0.5 ± 2.8	0.7 ± 2.9	0.7 ± 2.8	0.588
Patients with significant angular change, *n* (%)	10 (5.21%)	4 (3.88%)	1 (5.56%)	5 (7.81%)	0.551
Whole spine X-ray (standing), °	32.9 ± 7.7	32.6 ± 7.5	35.0 ± 8.9	32.7 ± 7.7	0.663
Imaging intervals, months					
Initial CT → Whole spine X-ray	1.8 ± 2.7	1.8 ± 2.8	1.7 ± 2.1	1.8 ± 2.7	0.973
Spine X-ray → 1st follow-up CT	6.0 ± 5.6	6.4 ± 5.7	5.0 ± 6.1	5.7 ± 5.4	0.516
1st follow-up CT → Last follow-up CT	34.2 ± 7.8	33.9 ± 8.5	35.6 ± 7.0	34.2 ± 6.9	0.701
Initial CT → Last follow-up CT	42.6 ± 6.3	42.6 ± 6.5	43.1 ± 4.8	42.5 ± 6.5	0.934

Data are expressed as mean ± standard deviation (SD) or number (%). Group comparisons at each time point were performed using one-way analysis of variance or Kruskal–Wallis test, as appropriate. Abbreviations: ANOVA = analysis of variance; BCS = breast-conserving surgery; CT = computed tomography; IRB = immediate reconstruction after mastectomy; MA = mastectomy alone; SD = standard deviation.

## Data Availability

The data presented in this study are available upon request from the corresponding author. The data are not publicly available due to privacy restrictions involving patient information.
